# Association between oncogenic status and risk of venous thromboembolism in patients with non-small cell lung cancer

**DOI:** 10.1186/s12931-018-0791-2

**Published:** 2018-05-09

**Authors:** Feifei Dou, Huiqiao Li, Min Zhu, Lirong Liang, Yuan Zhang, Jiawen Yi, Yuhui Zhang

**Affiliations:** 1 Department of Respiratory and Critical Care Medicine, Beijing Chao-Yang Hospital, Capital Medical University, Beijing Institute of Respiratory Medicine, Beijing, 100020 China; 2 Department of Clinical Epidemiology, Beijing Chao-Yang Hospital, Capital Medical University, Beijing, 100020 China

**Keywords:** Non-small cell lung cancer, Venous thromboembolism, Mutation, Oncogene, Epidermal growth factor receptor, Kitten rat sarcoma

## Abstract

**Background:**

Preclinical data suggest that oncogene (EGFR and KRAS) events regulate tumor procoagulant activity. However, few studies have prospectively investigated the clinical relevance between the presence of EGFR or KRAS mutations and occurrence of venous thromboembolism(VTE) in patients with non-small cell lung cancer (NSCLC).

**Methods:**

A total of 605 Chinese patients with newly diagnosed NSCLC were included and were followed for a maximum period of 4.5 years. EGFR and KRAS mutations were determined by amplification refractory mutation system polymerase chain reaction method at inclusion. The main outcome was objectively confirmed VTE.

**Results:**

Of the 605 patients, 40.3% (244) had EGFR mutations and 10.2% (62) of patients had KRAS mutations. In multivariable analysis including age, sex, tumor histology, tumor stage, performance status, EGFR and KRAS status, EGFR wild-type (sub-distribution hazard ratio 1.81, 95% confidence interval 1.07–3.07) were associated with the increased risk of VTE. In competing risk analysis, the probability of developing VTE was 8.3% in those with and 13.2% in those without EGFR mutations after 1 year; after 2 years, the corresponding risks were 9.7 and 15.5% (Gray test *P* = 0.047).

**Conclusions:**

EGFR mutations have a negative association with the risk of VTE in Chinese patients with NSCLC.

## Background

Venous thromboembolism (VTE) is a frequent complication seen in patients with non-small cell lung cancer (NSCLC) [1–3] and is associated with poor quality of life and worse prognosis [4–6]. The mechanisms responsible for VTE in patients with cancer, however, are not fully understood.

Tissue factor (TF) is the primary cellular initiator of blood coagulation and a modulator of angiogenesis and metastasis in cancer [7, 8]. Tumor cells frequently overexpress TF and spontaneously release TF-positive microparticles into the blood, which are small membrane vesicles that are highly procoagulant [9–11]. Further, preclinical data suggest that genetic links (activation of oncogenes such as EGFR, RAS or MET, and inactivation of tumor suppressor genes such as p53 or PTEN) directly induce the expression of genes controlling hemostasis (such as TF gene), which can extend systemically hypercoagulability and cancer progression [12–19]. Still, little is known about the clinical relevance of these links between oncogenic status and the risk of VTE in NSCLC.

Both EGFR and KRAS mutations are most frequent oncogenic driver mutations for NSCLC [20, 21]. The frequency of oncogenic mutations was associated with race [22, 23]. Asian patients had the relatively higher rate of EGFR mutations, but the lower rate of KRAS mutations than Caucasian [24, 25]. In the prospective observational study, we examined associations between the presence of EGFR or KRAS mutations and occurrence of VTE in Chinese patients with newly diagnosed NSCLC.

## Methods

### Study populations

Consecutive patients with newly diagnosed NSCLC between May 2012 and May 2017 who met the following inclusion criteria were included in the prospective observational study: histological confirmation of diagnosis; identification of EGFR and KRAS gene mutations; willingness to participate; and provided written informed consent. The exclusion criteria were as follows: any surgery, chemotherapy, or radiotherapy within the past 3 months before recruitment;a history of VTE (VTE diagnosis at least 3 months prior to recruitment) and the continuous use of anticoagulant drugs. The patients were followed up prospectively for a maximum 4.5 years’ observation period until the occurrence of death, loss of follow-up, withdrawal of consent, or the censure date (July 1, 2017).

### Diagnosis and classification of VTE

All the included patients were instructed about the symptoms of VTE and requested to report when such symptoms occurred, but no active screening for VTE was conducted. In case of symptoms, objective imaging methods were used to confirm or exclude the diagnosis of VTE. Deep vein thrombosis (DVT) events were confirmed by venous ultrasound imaging or a computed tomography venous angiogram. Pulmonary embolism (PE) events were confirmed by a computed tomography pulmonary angiogram or a ventilation-perfusion scan (if patients had renal insufficiency or allergy to contrast). In patients who had died during follow-up, death certificates and autopsy-reports, if available, were reviewed to establish or exclude the diagnosis of fatal PE or VTE. Then, all VTE events were presented to an independent adjudication committee including experts in the fields of angiology and radiology. The adjudication committee confirmed or excluded the diagnosis. In addition, patients with no symptoms of VTE underwent clinical surveillance every 2 or 3 months depending on the therapy. Accidentally detected VTE was considered as an event, if the committee determined that the event was of clinical significance.

### Laboratory methods

At the time of study entry, DNA of tumor tissues was screened for gene mutations. Tumour biopsy specimens were formalin-fixed and paraffin-embedded (FFPE) for histology and mutation analysis. Pathological assessment of serial FFPE tissue sections and clinical disease staging was according to the 2004 World Health Organization classification guidelines and the TNM staging system of the International Association for the Study of Lung Cancer (version 7). Isolation of genomic DNA from FFPE tissue for amplification refractory mutation system polymerase chain reaction (ARMS-PCR) mutation analysis was performed using the FFPE DNA Kit and DNA purification spin columns (Beijing ACCB Biotech Ltd). ARMS-PCR for tissue mutation detection was performed using the Human EGFR and KRAS Gene Mutations Fluorescence Polymerase Chain Reaction Diagnostic Kit (Beijing ACCB Biotech Ltd). Three-step PCR cycling was performed on the real-time Mx3000P instrument (Agilent, Santa Clara, CA, USA) with the following settings: 95 °C for 10 min, 40 cycles of 95 °C for 15 s, and 60 °C for 1 min. Mutation detection levels were assessed by threshold cycle (Ct) values with strong positives (Ct < 35) equivalent to mutation levels of > 5%, and weak positives (35 ≤ Ct < 38) equivalent to levels of 1 to 5%. Negative samples were defined with a Ct value ≥38.

### Statistical methods

Continuous variables were described by median and interquartile range. For the categorical variables, the percentages of patients in each category were calculated. The clinical characteristics were compared between subgroups of patients with and without VTE using the chi-square test or Fisher’s exact test, as appropriate. Fine-Gray regression analyses were used for calculating the risk factors of VTE. A competing risk analysis was performed to determine the cumulative incidence of VTE with death considered a competing event. Grey’s test was used to identify statistically significant differences between patients with different statuses of oncogenic mutations. A value of *P* < 0.05 was considered statistically significant. R statistical software was used to perform the competing risk analysis and Fine-Gray regression analyses. SPSS statistical software (Version 22.0; IBM, Armonk, NY, USA) was used for all other analyses. The study was reviewed by an epidemiologist and conformed to all the items of the Strobe statement.

## Results

### Patient characteristics

**Fig. 1:**
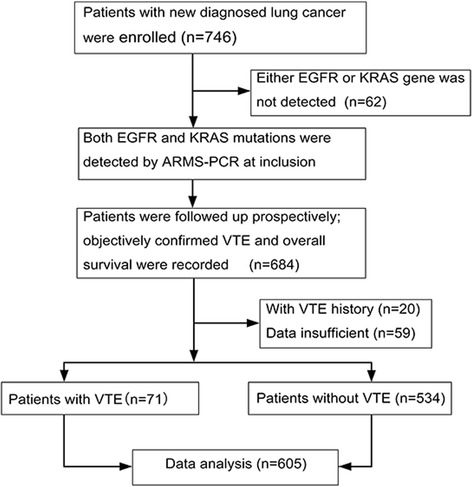
Study flow diagram

A total of 746 consecutive patients with newly diagnosed NSCLC were enrolled in this study. Sixty two patients were excluded because they lacked adequate material for detection of EGFR and KRAS mutations. Twenty patients were excluded because they had a history of DVT or PE more than 3 months before recruitment. Another 59 patients were excluded because they lacked complete information on follow-up. In the end, 605 eligible patients were included in our study (Fig. 1).

The 605 included NSCLC patients had a median age of 62.0 years, and 58.2% of the patients were males. The population consisted of 471 patients with adenocarcinoma (77.9%) and 134 patients with nonadenocarcinoma (22.1%). There were 417 stage IV patients (68.9%) with distant metastases, of which 7 were symptomatic brain metastases, and 17 were asymptomatic brain metastases. Furthermore, 24 of the patients had symptomatic bone metastases and 117 of the patients had asymptomatic bone metastases. An additional 29 patients presented with both brain and bone metastases, 13 of which were symptomatic. Patients with PS = 2–3 were considered to be a particularly frail group, with a total of 181 patients. Among them, there were 162 patients with PS = 2, 19 patients with PS = 3. The baseline demographic and clinical characteristics of the investigated study population are listed (Table 1).

**Table 1:** Baseline demographic and clinical characteristics of the study population

Characteristic	All patients	Patients with VTE
	(*n* = 605) (%)	(*n* = 71) (%)
Median age, years	62	60
25th–75th percentile	55–69	51–69
< 60	237 (39.2)	32 (45.1)
≥ 60	368 (60.8)	39 (54.9)
Sex		
Male	352 (58.2)	40 (56.3)
Female	253 (41.8)	31 (43.7)
ECGO PS		
0–1	424 (70.1)	41 (57.7)
2–3	181 (29.9)	30 (42.3)
Tumor histology		
Adenocarcinoma	471 (77.9)	62 (87.3)
Non-adenocarcinoma	134 (22.1)	9 (12.7)
Squamous cell carcinoma	125 (20.7)	8 (11.3)
Other NSCLC	9 (1.4)	1 (1.4)
Tumor Stage		
Localized	137 (22.6)	16 (22.5)
Distant metastasis	468 (77.4)	55 (77.5)
EGFR gene		
Wild	361 (59.7)	49 (69.0)
Mutated	244 (40.3)	22 (31.0)
KRAS gene		
Wild	543 (89.8)	61 (85.9)
Mutated	62 (10.2)	10 (14.1)
Treatment during observation period		
Other treatment without TKI	220 (36.4)	27 (38.0)
Other treatment with TKI	96 (15.9)	13 (18.3)
TKI alone	141 (23.3)	11 (15.5)
Chemotherapy alone	148 (24.5)	20 (28.2)

Abbreviations: *ECOG* Eastern Cooperative Oncology Group, *PS* performance status, *EGFR* epidermal growth factor receptor, *KRAS* kitten rat sarcoma, *NSCLC* non-small cell lung cancer, *TKI* Tyrosine Kinase Inhibitor

### Development of VTE

A total of 243 of the 605 patients (40.2%) died during follow-up, and 362 were alive at the censure date (59.8%). Of those who died during follow-up, 32 of them had localized disease while the other 211 had distant metastasis at the time of recruitment. Overall, 71 of the 605 patients (11.7%) experienced a VTE event from date of diagnosis to date of last follow-up. Of those, in 44 patients (7.3%) DVT alone developed (including the lower extremity, upper extremity, neck, or pelvis DVT), PE alone developed in 7 patients (1.1%, including 3 fatal PE), and both DVT and PE developed in 20 patients (3.3%) (Table 2).

**Table 2:** Incidence and types of VTE in NSCLC

Type/Site of VTE	No. of patients (%)
Total episodes	71 (11.7)
DVT alone	44 (7.3)
Upper extremity and neck	10 (1.6)
Lower extremity and pelvis	32 (5.3)
Upper extremity and lower extremity	2 (0.3)
PE alone	7 (1.1)
Segmental/subsegmental	4 (0.6)
Above segmental	3 (0.5)
DVT and PE combined	20 (3.3)
Lower extremity, pelvis DVT and segmental PE	13 (2.1)
Upper extremity, pelvis DVT and above segmental PE	1 (0.2)
Lower extremity, pelvis DVT and above segmental PE	4 (0.6)
Upper extremity, lower extremity DVT and segmental PE	2 (0.3)

Abbreviations: *DVT* deep vein thrombosis, *NSCLC* non-small cell lung cancer, *PE* pulmonary embolism, *VTE* venous thromboembolism

### Mutations and risk of VTE

Both EGFR (exons 18, 19, 20, and 21) and KRAS (codons 12 and 13) mutations were determined by ARMS-PCR at inclusion. Of the 605 patients, 40.3% (244) had EGFR mutations and 10.2% (62) of patients had KRAS mutations. The types of EGFR and KRAS mutations and frequency of VTE are listed (Table 3). There was no significant association between mutation type and risk of VTE (Table 3).

**Table 3:** Types of EGFR and KRAS mutations and frequency of VTE

Mutation type	Number	VTE
	(*n* = 306) (%)	(*n* = 32) (%)
EGFR exon 18 mutation only	3 (1.0)	0 (0.0)
EGFR exon 19 deletion only	106 (34.6)	9 (28.1)
EGFR exon 20 T790 M only	0 (0.0)	0 (0.0)
EGFR exon 20 S768I only	4 (1.3)	0 (0.0)
EGFR exon 20 insertion only	11 (3.6)	0 (0.0)
EGFR exon 21 L858R only	106 (34.6)	12 (37.5)
EGFR exon 21 L861Q only	7 (2.3)	0 (0.0)
EGFR exon 18 + exon 21 L861Q	1 (0.3)	0 (0.0)
EGFR exon 18 + exon 20 S768I	2 (0.6)	1 (3.1)
EGFR exon 19 + exon 21 L861Q	1 (0.3)	0 (0.0)
EGFR exon 19 + exon 20 T790 M	1 (0.3)	0 (0.0)
EGFR exon 20 T790 M + exon 21 L858R	1 (0.3)	0 (0.0)
EGFR exon 20 S768I + exon 21 L858R	1 (0.3)	0 (0.0)
KRAS codon G12C(34G > T) only	19 (6.2)	5 (15.6)
KRAS codon G12S(34G > A) only	2 (0.6)	2 (6.3)
KRAS codon G12R(34G > C) only	5 (1.6)	0 (0.0)
KRAS codon G12 V(35G > T) only	14 (4.6)	3 (9.4)
KRAS codon G12D(35G > A) only	12 (3.9)	0 (0.0)
KRAS codon G12A(35G > C) only	9 (2.9)	0 (0.0)
KRAS codon G13D(38G > A) only	0 (0.0)	0 (0.0)
KRAS codon G12R(34G > C) + codon G12D(35G > A)	1 (0.3)	0 (0.0)

Abbreviations: *EGFR* epidermal growth factor receptor, *KRAS* kitten rat sarcoma

**Fig. 2:**
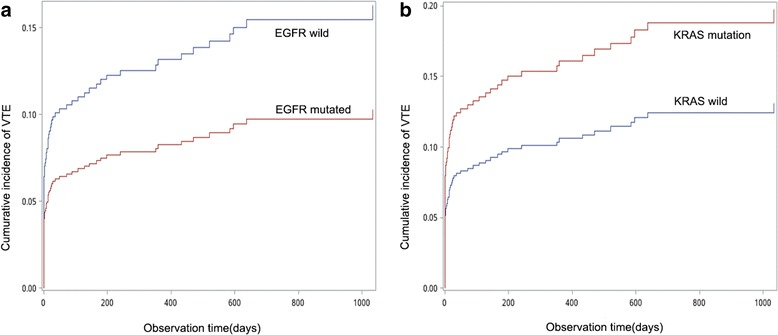
Competing risk analysis. **a**. The cumulative incidence of venous thromboembolism (VTE) in non-small cell lung cancer (NSCLC) patients with and without EGFR mutations (*P* = 0.047) assessed by the competing risk analysis. **b**. The cumulative incidence of venous thromboembolism (VTE) among non-small cell lung cancer (NSCLC) patients with and without KRAS mutations (*P* = 0.180) assessed by the competing risk analysis

Of the 244 patients with EGFR mutations, VTE developed in 22 (9.0%), whereas VTE developed in 49 of 361 patients (13.6%) with EGFR wild-type. After 1 year, the probability for development of VTE was 8.3% in those with and 13.2% in those without EGFR mutations; after 2 years, the corresponding risks were 9.7 and 15.5% (Gray test *P* = 0.047) (Fig. 2a). Moreover, of the 62 patients with KRAS mutated type, VTE developed in 10 (16.1%), whereas VTE developed in 61 of 543 patients (11.2%) with KRAS wild-type. After 1 year, the probability for development of VTE was 16.1% in those with and 10.6% in those without KRAS mutations; after 2 years, the corresponding risks were 18.8% and 12.4%, respectively (Gray test *P* = 0.180) (Fig. 2b).

Subsequently, we performed a Fine-Gray regression model that included the age, sex, different tumor histology (adenocarcinoma vs. nonadenocarcinoma), tumor stage (localized stage vs. distant metastasis), Eastern Cooperative Oncology Group performance status (0–1 vs. 2–3), EGFR and KRAS mutations (mutated vs. wild-type) to identify the factors associated with VTE. Adenocarcinoma [sub-distribution hazard ratio (SHR) 2.40, 95% confidence interval (CI) 1.11–5.19,*P* = 0.027], poor performance status (SHR 1.91, 95% CI 1.18–3.09, *P* = 0.008), and EGFR wild-type (SHR 1.81, 95% CI 1.07–3.07, *P* = 0.028) were associated with an increased risk of VTE. Age, sex, KRAS gene and tumor stage were not associated with the development of VTE (Table 4).

**Table 4:** Factors associated with increased risk of VTE in patients with NSCLC

Patients Group	SHR	95% CI	*P-value*
Tumor histology (%)			
Non-adenocarcinoma	1		
Adenocarcinoma	2.40	1.11–5.19	0.027
ECOG PS			
0–1	1		
2–3	1.91	1.18–3.09	0.008
EGFR gene			
Mutated	1		
Wild	1.81	1.07–3.07	0.028
Age			
≥ 60	1		
< 60	1.27	0.79–2.02	0.324
Sex			
Female	1		
Male	1.03	0.63–1.66	0.920
Tumor Stage			
Distant metastasis	1		
Localized	1.18	0.67–2.07	0.569
KRAS gene			
Wild	1		
Mutated	1.10	0.52–2.32	0.814

Abbreviations: *CI* confidence interval, *ECOG* Eastern Cooperative Oncology Group, *SHR* Sub-distribution hazard ratio, *NSCLC* non-small cell lung cancer, *PS* performance status, *VTE* venous thromboembolism

*The variables were entered into the Fine-Gray regression model and included age, gender, ECOG PS (0–1 vs. 2–3), EGFR (mutated vs. wild), KRAS (mutated vs. wild), tumor histology (adenocarcinoma vs. nonadenocarcinoma), and tumor stage (localized stage vs. distant metastasis). All variables were shown in the table

## Discussion

In the prospective observational study population of Chinese patients with newly diagnosed NSCLC, the presence of EGFR mutations might decrease the risk of VTE, whereas KRAS mutations were not significantly associated with VTE risk.

### EGFR status and risk of VTE

The assessment of whether oncogenic mutations affect the risk of thrombosis has been a focus of preclinical research and clinical study. Our study revealed that the risk of VTE was 1.81 (95% CI of 1.07 to 3.07) higher in patients with EGFR wild compared to those with EGFR mutated. Preclinical data showed that amplification of EGFR or mutated EGFR vIII induces the overexpression of TF by cancer cells [14, 15]. The increase in TF may constitute a direct link between thrombosis risk and oncogene expression in patients with cancers [8]. We assumed that EGFR mutations (exons 18, 19, 20, and 21) might decrease the expression of TF, which reduces tumor procoagulant activity and the incidence of VTE. However, previous three studies reported no association between EGFR gene status and VTE risk in patients with NSCLC [26–28]. The possible explanations of different findings were that previous studies used retrospective design and included patients from different race population.

### KRAS status and risk of VTE

Limited preclinical data suggest that KRAS mutational status of the tumor represents a plausible clinical link to systemic hypercoagulability in cancer patients [12, 13]. Conflicting results, however, have been reported for KRAS mutations in previous clinical studies. In our study, KRAS mutations were not independently associated with the risk of VTE in NSCLC patients. This finding was in agreement with some studies [26, 27]. However, the finding of our study was in disagreement with a published report on metastatic colorectal cancer [29]. This difference was likely because our study population consisted of NSCLC patients who had different biological characteristics. The risk of KRAS status may be weakened due to lower incidences of KRAS mutations in NSCLC than in metastatic colorectal cancer. Corrales-Rodriguez [28]found a correlation between KRAS mutation and increased risk of VTE among patients with NSCLC in a retrospective case-control study. The possible explanations of different findings were that our study had prospective design and investigated a Chinese population with lower KRAS mutations.

### Other factors and risk of VTE

The Fine-Gray regression analyses showed two additional factors related to the occurrence of VTE. The first factor is histological type. In our study, patients with adenocarcinoma had a 2.40-fold higher risk of VTE than patients with non-adenocarcinoma (95% CI of 1.11–5.19), which is in agreement with previous studies [30, 31]. Similar results were observed in studies of PE in patients with lung cancer [2, 32]. The second factor is performance status. A previous study showed that poor performance status was correlated with increased risk of thrombosis [33]. Similarly, our study revealed a 1.91-fold higher risk of VTE in patients with poor performance status compared to those with better performance status (95% CI of 1.18–3.09), which is in agreement with the previous study. We found no association between age, sex or tumor stage with the occurrence of VTE.

### Limitations

Our study has several limitations. Although we included a large number of NSCLC patients in this prospective cohort study, the number of patients with KRAS mutations is still relatively low. Moreover, we followed our patients carefully at regular intervals and concentrated on the most clinically relevant symptomatic VTE events in our study, but we did not screen for VTE, which possibly missed asymptomatic VTE. Finally, only common KRAS (codons 12 and 13) mutations were detected, while rare KRAS mutations might be missed.

## Conclusions

The presence of EGFR mutations was associated with decreased risk of VTE, and the analysis of EGFR status might be helpful for identifying VTE risk for NSCLC. Because the number of patients with KRAS mutations is relatively low, the link between KRAS mutations and VTE needs to be elucidated in further large-scale prospective studies.

## Abbreviations

ARMS-PCR: Amplification refractory mutation system polymerase chain reaction
CI: Confidence interval
DVT: Deep vein thrombosis
ECOG: Eastern cooperative oncology group
EGFR: Epidermal growth factor receptor
FFPE: Formalin-fixed and paraffin-embedded
HR: Hazard ratio
KRAS: Kitten rat sarcoma
NSCLC: Non-small cell lung cancer
PE: Pulmonary embolism
PS: Performance status
TF: Tissue factor
VTE: Venous thromboembolism
